# Mapping of clinical research on artificial intelligence in the treatment of cancer and the challenges and opportunities underpinning its integration in the European Union health sector

**DOI:** 10.1093/eurpub/ckac016

**Published:** 2022-03-03

**Authors:** Elena-Ramona Popescu, Marius Geantă, Angela Brand

**Affiliations:** Center for Innovation in Medicine, Bucharest, Romania; Center for Innovation in Medicine, Bucharest, Romania; KOL Medical Media, Bucharest, Romania; United Nations University—Maastricht Economic and Social Research Institute on Innovation and Technology, Maastricht, The Netherlands; United Nations University—Maastricht Economic and Social Research Institute on Innovation and Technology, Maastricht, The Netherlands; Faculty of Health, Medicine and Life Sciences, Maastricht University, Maastricht, The Netherlands; Department of Public Health Genomics, Manipal School of Life Sciences, Manipal Academy of Higher Education, Manipal, India; Dr. TMA Pai Endowment Chair in Public Health Genomics, Manipal School of Life Sciences, Manipal Academy of Higher Education, Manipal, India

## Abstract

**Background:**

Although current efforts are made to diminish the incidence and burden of disease, cancer is still widely identified late at stage. This study aims to conduct a systematic review mapping the existent and emerging clinical research on artificial intelligence (AI) in the treatment of cancer and to underpin its integration challenges and opportunities in the European Union (EU) health sector.

**Methods:**

A systematic literature review (SLR) evaluating global clinical trials (CTs; published between 2010 and 2020 or forthcoming) was concluded. Additionally, a horizon scanning (HS) exercise focusing on emerging trends (published between 2017 and 2020) was conducted.

**Results:**

Forty-four CTs were identified and analyzed. Selected CTs were divided into three research areas: (i) potential of AI combined with imaging techniques, (ii) AI’s applicability in robotic surgery interventions and (iii) AI’s potential in clinical decision making. Twenty-one studies presented an interventional nature, nine papers were observational and 14 articles did not explicitly mention the type of study performed. The papers presented an increased heterogeneity in sample size, type of tumour, type of study and reporting of results. In addition, a shift in research is observed and only a small fraction of studies were completed in the EU. These findings could be further linked to the current socio-economic, political, scientific, technological and environmental state of the EU in regard to AI innovation.

**Conclusion:**

To overcome the challenges threatening the EU’s integration of such technology in the healthcare field, new strategies taking into account the EU’s socio-economic and political environment are deemed necessary.

## Introduction

Cancer constitutes the second leading cause of mortality worldwide. In 2018, the overall number of deaths was estimated to be 9.6 million individuals.^1^ For instance, despite only representing 9% of the global population, Europe accounted for 23.4% of global cancer cases and 20.3% of total deaths.^2^ By the year 2040, projections estimate an increase of 47% compared with the number of cases in 2020.^3^ Such occurrence is strongly associated with causes such as alcohol consumption, tobacco, unhealthy diets and reduced physical activity.^4^^,^^5^ Limited access to treatment facilities, reduced availability of diagnosis resources and/or socio-economic inequalities underlie further significant differences in survival rates across European Member States (MS).^6^

To combat the effects of the disease in the European Union, the Commission has proposed a new cancer plan. The Europe’s Beating Cancer Plan aims to enhance cancer survival rates by tackling the entire disease pathway while, at the same time, significant inequalities between and within MS are reduced.^4^ At present, cancer innovation has mainly been redirected towards improving disease prevention and diagnosis. However, often, cancer is still identified late at stage leading to the need of new treatment strategies. For instance, survival rates for liver cancer vary from 20.7% in Belgium to only 4.2% in Estonia, whereas the mortality rate for cervical cancer in Romania accounts for 14.2% compared with the European Union (EU) average of 3.7%.^7^^,^^8^ More information on the initiatives and actions proposed under the new Europe’s Beating Cancer Plan can be found in Supplementary table A1.

The new Cancer Plan will concentrate on all key phases of the disease and will complement Member States’ already existing national cancer agendas. The plan further recognizes the growing impact of fields such as high-performance computing, big data and AI.^9^ Currently, the early diagnosis of the disease, the classification of cancer patients in high- or low-risk subgroups and further prognosis are critical for accurate patient management.^10^

As the new Cancer Plan recognizes the potential of big data and AI, as well as, the need for new therapeutic strategies, this study aims to map the clinical applicability of AI in improving cancer treatment and to analyze the challenges and opportunities that may underpin its integration across the EU.

## Methods

### Systematic literature review

A comprehensive and computerized search of the following databases was performed: (i) Public Medline,^11^ (ii) Clinical Trials,^12^ (iii) Global Clinical Trials.^13^ For the scope of this research, only clinical trials (CTs) were considered and analyzed. CTs were selected as both the efficacy of AI and its safety were generally presented.^14^ Several AI technologies such as machine learning (ML), deep learning (DL) and neural network (ANNS) were included. In addition, studies must have been in English, published between 2010 and 2020 or estimated to be published in the forthcoming months or years. The following search strings were employed: (‘artificial intelligence’ OR ‘machine learning’ OR ‘deep learning’ OR ‘neural network*) AND (‘cancer’ OR ‘tumour*’ OR ‘malignan*’) AND (‘treatment’ OR ‘therap*’) AND (‘effectiveness’ OR ‘potential’).

The following inclusion criteria were established: (i) stage I to IV—completed, active or emerging—randomized and non-randomized CTs conducted in cancer patients with no restriction of age and sex, (ii) CTs exploring physical tumours with AI, ML, DL and/or ANNs interventions and (iii) CTs assessing the effectiveness and/or potential of AI technology.

Studies were excluded on the basis that they were (i) focusing on patients suffering from other diseases, (ii) utilizing AI, ML, DL and ANNs for other interventions (i.e. behavioural assessments of cancer patients), (iii) other outcomes were assessed (i.e. psychological factors) and (iv) CTs were not published or registered in English and dated before 2010.

### Horizon scanning

The horizon scanning (HS) exercise, defined as the ‘systematic outlook to detect early signs of potentially important developments’, was conducted according to the STEEPS framework. STEEPS is a foresight tool enabling the scanning of social, technological, environmental, economic, political and scientific factors that may modify and alter the EU’s implementation and integration of AI in cancer treatment. Such analysis facilitates the identification of possible favourable circumstances and early signals of threats.^15^ The specific search strategy was based on an open approach including the search strings employed during the SLR and further extended by the following concepts: ‘changes’, ‘challenges’ and ‘opportunities’.

A search of the following databases was performed: (i) Public MedLine and (ii) ScienceDirect databases. Additionally, grey literature from European international institutions, governmental agencies and publicly available policy reports was extracted. Selected studies must have been in English, published between 2017 and 2020—allowing, therefore, for the identification of most current changing trends, challenges and arising threats—and located in European Member States.

### Data analysis

#### Systematic literature review

The following information was extracted from the studies included in the SLR: (i) author, year, location, (ii) title of the study, (iii) population, (iv) intervention(s) and (v) outcomes (see Supplementary table B1). Studies matching the inclusion criteria and selected were structured into the following research themes: (i) AI and Cancer Imaging Implications, (ii) AI and Robotic Surgery and (iii) AI and Clinical Decision Making.

#### Horizon scanning

The results obtained from the HS exercise were analyzed and categorized according to the STEEPS framework.

## Results

### Study identification and selection

**Figure 1:**
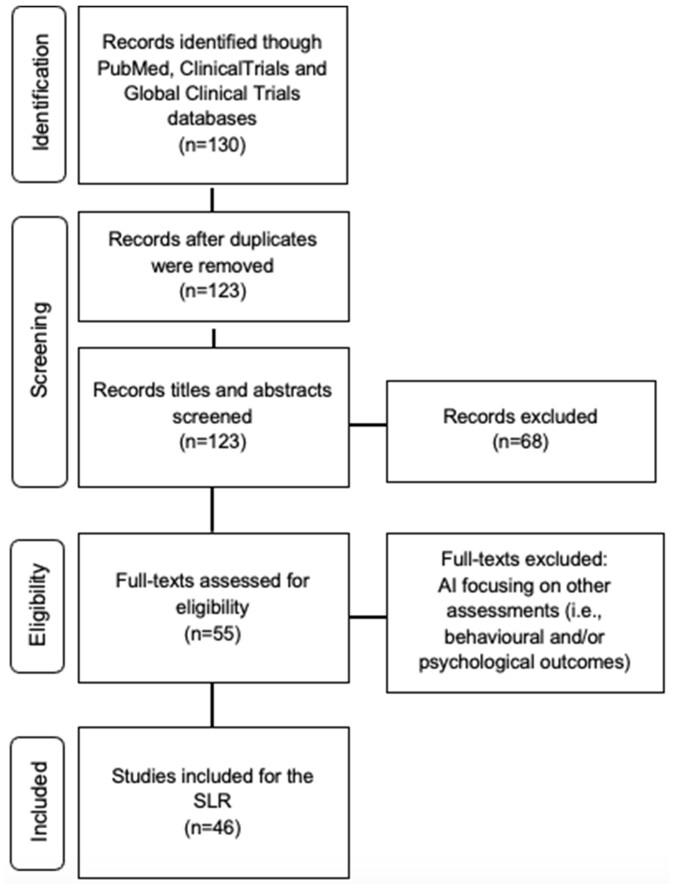
The Prisma flow chart representing the study selection process for the systematic literature review. Adapted from Moher et al^16^

Throughout the systematic literature review, the searching databases identified a total of 130 titles. The studies were exported to Mendeley’s reference manager software and duplicates (seven) were consequently removed. The remaining 123 CTs were screened based on title and abstract, resulting in the further removal of 68 identified studies. The full text of 55 CTs was carefully examined for eligibility. After examining the full texts diligently, 10 clinical studies were further excluded as other factors such as psychological outcomes were reported. Finally, 44 CTs—completed, active and emerging—were included and analyzed (figure 1).

### AI and clinical imaging implications

Articles exploring AI’s implication in clinical imaging were published between 2010 and 2019 or expected to be finalized in the forthcoming months and years (Supplementary table C1).^17–29^

Completed studies were performed in the USA (*n* = 2), China (*n* = 1), Iran (*n* = 1) and only one study was identified as multicentric. The number of participants ranged from 11 to 400 patients and the main areas of study were represented by rectal cancer (*n* = 1), non-squamous cell carcinoma (*n* = 1), oesophageal squamous cell carcinoma (*n* = 1), breast cancer (*n* = 1) and lung cancer (*n* = 1). From these studies, only two papers reported the 95% confidence interval (CI) and explicitly mentioned the average follow-up period or follow-up checkpoints.

Most of these studies focused on exploring the potential of AI on patients’ radiomic features obtained from images acquired with magnetic resonance imaging, positron imaging tomography and/or computed tomography scans. The authors aimed to develop effective bioimaging markers to predict intervention outcomes and treatment failure by the stratification of patients. For instance, the support vector machine (SVM) classifier was predominantly used in three out of five papers.^17^^,^^19^^,^^20^ Other classifiers used included the neural networks (NN), Bayesian network (BN), k-neighbour classifier (KNN), random forest (RF), logistic regression and learning machine (ELM). Shayesteh et al. reported accuracy in predictive performance of 97.8% and 92.8% in testing and 95% and 90% in validation tests, using four classifiers (SVM.NN.BN.KNN) to predict neo-adjuvant chemoradiotherapy response.^17^ Xiong et al. presented the use of an RF classifier with an accuracy of 93.3%, specificity of 95% and a sensitivity of 85.7% in stratifying patients with chances of local failure following concurrent chemoradiotherapy.^19^ Zhao et al., on the other hand, aimed to classify breast cancer patients into prone or supine classes (preferred treatment position) using a two-stage SVM classifier. Overall sensitivity and specificity were found to be 90.4% and 99.3%, respectively.^20^

These results demonstrate that AI applied on imaging scans is both highly sensitive and specific.

In addition, eight forthcoming papers in this area are estimated to finalize between late 2021 and 2025.^22–29^ Identified papers are located in China (*n* = 8), Canada (*n* = 1) and France (*n* = 1). The main areas of study targeted are hepatocellular carcinoma (*n* = 1), rectal cancer (*n* = 1), prostate cancer (*n* = 1), gliomas (*n* = 2), cervical cancer (*n* = 1), breast cancer (*n* = 1) and colorectal cancer (*n* = 1). The number of estimated patients ranges from 34 to 1200 individuals and only one study mentions the targeted follow-up period. Three papers are reported as interventional studies (one randomized CT) and four articles are of observational nature. Seven out of eight of these studies will focus on exploring the predicted patient outcome response to different therapeutic interventions.^22–25^^,^^27–29^ Only one study focuses on combining DL and image recognition with big data analysis to define the characteristics of molecular subsets of gliomas.^26^ This has great potential in clinical research of glioma diagnosis, prognosis and treatment options.

### AI and robotic surgery

Articles analyzing the potential of AI combined with robotic surgery, as presented in Supplementary table C2, were published between 2010 and 2019.^30–49^

Studies were performed in the USA (*n* = 8), Korea (*n* = 2), The Netherlands (*n* = 2), Switzerland (*n* = 1), Italy (*n* = 1), Spain (*n* = 1), France (*n* = 1), India (*n* = 1) and three CTs were identified as multicentric. The number of participants ranged from one single case study to 265 cancer patients and the main areas of study were concentrated on prostate cancer (*n* = 8), bladder cancer (*n* = 3), rectal cancer (*n* = 3), colorectal cancer (*n* = 1), oropharyngeal cancer (*n* = 1), adrenal cancer (*n* = 1), gastrointestinal cancer (*n* = 1), renal cancer (*n* = 1) and intrathoracic oesophageal cancer (*n* = 1). Most of the CTs performed random allocation of patients (*n* = 9) and only three papers reported the 95% CI, whereas 13 articles mentioned the average follow-up period or follow-up checkpoints. Ten articles were identified as intervention studies, whereas only one study was observational. Only five papers were published after 2015.

Five completed studies mainly focused on comparing the laparoscopic radical proctectomy (LRP) with the robot-assisted radical proctectomy (RARP) technique in prostate cancer patients, implementing the innovative Revo-I robotic surgical system and exploring the emerging innovation in RARP.^30–34^ Studies were in general consistent with their results and no significant differences were found between LRP and RARP in light of operating time, blood loss and transfusion rates among other variables. However, as suggested by Asimakopoulos et al., Di Pierro et al. and Porpiglia et al., RAPRP performed slightly better in terms of capability of intercourse, erectile function, recovery of continence and potency. Three identified randomized CTs compared the open radical cystectomy technique with a robotic cystectomy procedure.^30^^,^^32^^,^^33^ All three studies concluded that robotic cystectomy was safe and compared favourable in some perioperative parameters. Main outcomes assessed across these studies were estimated blood loss, estimated length of stay and postoperative complications. Park et al. explored the therapeutic applicability, safety and effectiveness of a reverse hybrid robotic laparoscopic rectal resection system and Colombo et al. investigated the effects of right total mesorectal excision compared with left total mesorectal excision.^43–44^ Five additional studies exploring the application of AI in robotic surgery focusing on the treatment of oropharyngeal cancer, adrenal cancer, gastrointestinal cancer and renal cancer were concluded.^45–49^

#### AI and clinical decision making

Completed articles exploring AI’s applicability in clinical decision making were published between 2012 and 2020 (Supplementary table C3).^50–60^

Only one paper was published before 2018. Studies were performed in China (*n* = 2), Taiwan (*n* = 1), The Netherlands (*n* = 1), USA (*n* = 1), Japan (*n* = 1) and one study was identified as multicentric. The number of participants ranged from 29 to 1742 individuals and main areas of investigation focused on leukaemia (*n* = 2), colorectal cancer (*n* = 1), prostate cancer (*n* = 1), head and squamous carcinoma (*n* = 1) and nasopharyngeal carcinoma (*n* = 1). From these studies, six papers reported 95% CI and only four papers mentioned the average follow-up period or follow-up checkpoint times. Three articles were interventional studies, one paper was a randomized CT, whereas only one study was reported as observational.

Ko et al. concluded a randomized CT aiming to analyze the effectiveness of AI to complete a multiflow cytometry analysis.^50^ This is of particular interest as such a technique helps identify residual anomalies that may have been left or developed. The authors were able to develop promising algorithms with accuracies ranging from 84.6% to 92.4% and with clinical significance (*P* < 0.00001). On the other hand, Wagner et al. trained an artificial neural network to identify a predictive outcomes biomarker.^52^ The applied artificial neural network identified a parsimonious three-gene expression signature comprising CALCRL, CD109 and LSP2, which was predictive of event-free survival and overall survival. The biomarker had the ability to stratify patients in distinct subgroups based on their survival probabilities. Similarly, Wan et al. and Skrede et al. developed a stratification biomarker able to classify patients at risk of nasopharyngeal carcinoma and stage I and stage II colorectal cancer patients, respectively.^51,55^ Wan et al. testes a set of SVM models and reported a sensitivity ranging from 84 to 88% and a specificity ranging from 81.9 to 94.5%, whereas Skrede et al. obtained a clinical significance of *P* < 0.00001.

Dai et al. and Zhong et al. further explored the potential of AI to analyze molecular data and determine a predictive biomarker for treatment outcomes. These CTs focused on prostate cancer patients treated with finasteride and head and neck squamous carcinoma patients treated with taxane, cisplatin and 5-fluorouracil chemotherapy, respectively.^53,54^ The study concluded by Zhong et al. presents a sensitivity of 88% and a specificity of 88.9% in TPF-sensitive patients and a sensitivity of 75% and a specificity of 100 in TPF non-sensitive patients. Results in both studies suggested a statistically significant potential of AI to perform such activities.

On the other hand, four forthcoming papers in the area are estimated to be finalized between late 2021 and 2022. One study does not mention estimated completion date and the location of the CT are found in China (*n* = 2), the USA (*n* = 1) and Taiwan (*n* = 1). The main focus areas will rely on colorectal cancer (*n* = 2), advanced malignancies (*n* = 1) and malignant polyps (*n* = 1) and the estimated number of patients range from 70 to 5000. Only one study reports a target follow-up period. In addition, two studies are interventional randomized CTs and two papers will be observational.

### AI integration in the EU healthcare system: challenges and opportunities

Three key sources included in this study were the following reports: (i) Transforming Healthcare with AI: The Impact on The Workforce and Organisations, (ii) European Artificial Intelligence (AI) Leadership, The Path for an Integrated Vision and (iii) Artificial intelligence in Medicine and Healthcare: Applications, Availability and Social Impact.^61–63^

**Figure 2:**
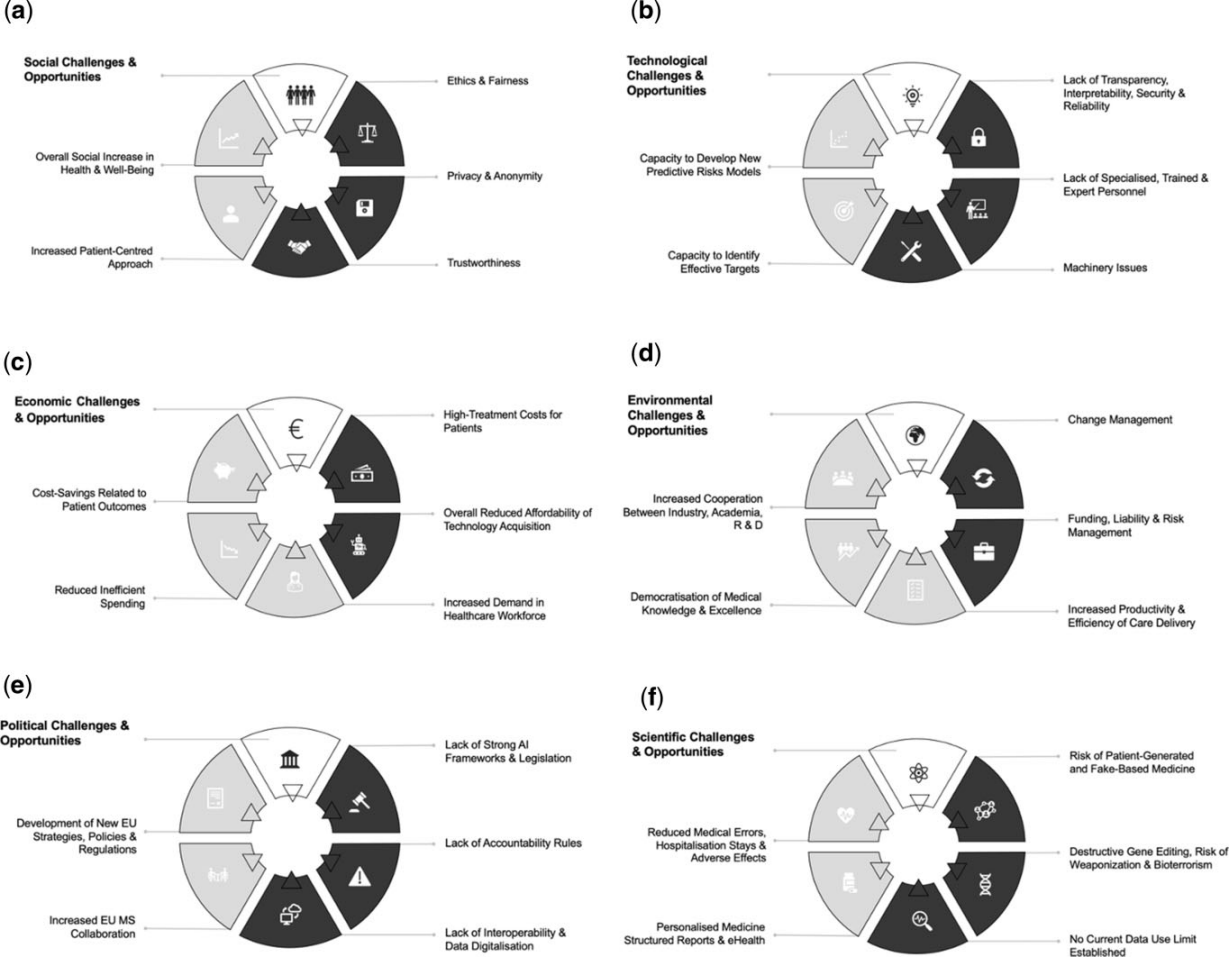
Chart representing AI challenges (black) and opportunities (grey) in EU health sector (a) social challenges and opportunities, (b) technological challenges and opportunities, (c) economic challenges and opportunities, (d) environmental challenges and opportunities, (e) political challenges and opportunities and (f) scientific challenges and opportunities for AI integration in the sector of health

Five overarching categories alternating both favourable outcomes and threats were determined for each field. The author established this distribution as the sum of all information retrieved from relevant sources (figure 2).

Current social challenges outweigh the potential opportunities that may arise regarding the integration of AI in the EU health sector. Some strong negative features linked to such a process include lack of fairness linked to bias embedded in AI inputs, inequal opportunities across Member States and current privacy and anonymity issues.^64^ Perceptions of fear and lack of trust usually related to little or no social knowledge or awareness about such technology further difficult the integration of AI in medical settings. However, AI’s capacity to enable a much more patient-centred approach would allow for a general increase in social health and well-being.^63,65^ AI has the potential to identify new targets that would lead to the development of accurate predictive risk models able to anticipate possible outcomes (figure 2a). Unfortunately, the current and persistent lack of technology, transparency, reliability, security and the lack of specialized and experienced personnel to rigorously interpret data and manage architecture-related issues causes difficulties to the overall technology integration and threatens its positive components (figure 2b).

Economic opportunities appear to outweigh challenges that may be currently present (figure 2c). For instance, the WHO estimates the creation of roughly 40 million new health sector jobs by 2030 worldwide.^62^ Recent studies also reflect that a 10% improvement in failure prediction before a CT could save around 100 million euros in development cost per drug.^65^ Thus, it reduces inefficient spending effectively. Additionally, payers could further benefit from cost-savings related to patient outcomes due to decreased medical costs and reduced losses from productivity. For example, breast cancer is estimated to save up to 74 million euros in the next 10 years.^63^ Notwithstanding, the integration of AI in healthcare will inevitably lead to increased costs in patient’s treatment. A current reduced affordability at organizational, regional and governmental levels also disrupts the equal acquisition of technology across and within Member States. These challenges act as a setback for the favourable opportunities.^63^

In addition, as represented in figure 2d, a positive increase in industry and stakeholder collaboration, an overall democratization of knowledge and sharing of expertise and, a general increase in productivity and efficiency in light of the delivery of care upon AI integration is expected. Nonetheless, factors such as reduced funding and/or liability combined with poor-risk and change management, act as barriers to research and development, as well as, to the innovation’s overall social integration. The current lack of strong governance frameworks and accountability rules, and challenges in data digitalization, may also threaten both the development of new strategies and subsequent collaboration between EU Member States. Thus, from a political perspective, there are currently more barriers to face than opportunities facilitating the AI integration (figure 2e). Similarly, the scientific field is further susceptible to a vast majority of threats regarding AI implementation. For instance, algorithms’ training based on fake-based medicine challenges accurate and non-biased evidence-based results. Fear towards the risk of weaponization strongly linked to no current limit of data use further complicates the social acceptance of such innovation in healthcare.^63,66^

In general terms, it concludes that both the environmental and economic fields display an overall beneficial aftermath. However, the challenges present in the social, political, scientific and technological spheres threaten the integration of AI and subsequently, the potential benefits that may arise.

## Discussion

At present, innovation in the cancer field has mainly been redirected towards improving disease prevention and diagnosis. However, often, cancer is still identified late at stage.^67^ For that reason, available effective treatment that would decrease mortality rates, individual adverse effects and the overall disease burden in society, results critical. For this study, AI has been selected as an emerging technology recognized by the Europe’s Beating Cancer Plan as potential in cancer management. To the authors’ knowledge, this is the first systematic review that maps the clinical applicability of AI in improving cancer treatment specifically, by identifying CTs and relevant areas of action. In general, results presented positive outcomes and no major complications were reported.

Large disparities between countries in regard to the number of CTs—completed or emerging—were observed. USA and China are leading competitors, whereas Europe is lagging behind. Notable differences also exist in light of the development of such CTs between EU Member States. France and The Netherlands are leading European countries funding such CTs, being followed by the UK and countries such as Denmark, Finland and Germany.

In addition, a shift paradigm in research is observed. Currently, both ongoing and emerging CT prioritize research focusing on AI imaging implications, as well as, its ability to facilitate clinical decisions. These studies are also increasing the number of patients involved and the number of studies performed. Latest CT focus on investigating AI’s capacity to forecast and assess outcomes instead of further exploring its potential in surgery. Despite AI in robotic surgery representing roughly 45% of all trials identified, 75% were published before 2015.

This shift can be linked to the important arising role of AI in the development of personalized medicine focusing on identifying effective targets and developing predictive risk models.^62^ In addition, these studies are aligned with current worldwide cancer research trends.^68^

Large disparities were present regarding the international geographic location of the identified CTs. Around 75% of the reported studies are located in non-European countries. These differences could be associated with the current lack of EU strong governance frameworks and policies. Further lack of a solid legislation can reinforce the sentiment of social untrustworthiness considering privacy and anonymity uncertainty.^63^ Additionally, lack of strong regulations may, in some cases, instigate the feeling of fear regarding no established limits of data use and the possibility of emerging bioterrorism scenarios.^59^ In parallel, it can also manage social levels of trust and fear by educating the population and increasing awareness concerning the innovation’s potential. For this, a trained specialized workforce shall be capacitated to reliably interpret data and deliver an accurate dissemination of results.^61^

Similarly, significant discrepancies were also observed within Member States. It is now clear that both research and innovation are essential; however, inequalities are still persistent. These differences may arise as a direct consequence of reduced Eastern affordability to acquire and integrate such technology and a reduced allocation of funding compared with Western countries.^69^ For instance, in 2016, The Netherlands—pioneer country alongside France in the development of EU CTs for cancer treatment—obtained roughly €55 million to fund emerging projects on personalized medicine, whereas United Kingdom, for example, received €239 million. On the other hand, countries such as Romania and Bulgaria were allocated four million euros and less than a million euros, respectively.^70^ These factors can lead to an increased lack of fair access to such innovative treatments.

Given the results exposed above, both national and EU institutions are encouraged to further identify existent challenges and knowledge gaps. Considering the new Europe’s Beating Cancer Plan, this study aimed to map the existent and emerging clinical research on AI in the treatment of cancer. However, due to the increased heterogeneity in sample size, type of tumour researched, type of study and differences in the reporting of results, the authors acknowledge the need for future studies focusing on homogenic cancer morphology and/or topography CTs and its link with current socio-economic and political EU environment.

### Limitations

Potential study limitations that should be considered are presented. For instance, (i) despite accounting for data and methods triangulation, this research is not supported by the revision, provided by an additional external investigator, of the sources chosen to be included. In addition, (ii) the key terms employed to design the search strategy and their consequent combination may have limited the number of sources identified. Furthermore, (iii) the number of studies identified may not represent the actual number of studies performed due to the possible non-mandatory registration of those in the databases of interest. Finally, (iv) this study did not integrate further expert input that would contrast and assess both the effectiveness and limitations of the concluded HS exercise.

## Conclusion

AI has the potential to improve cancer treatment by assisting current imaging techniques approaches or surgery procedures, as well as, by reinforcing clinical decision making. Unfortunately, its EU integration may face numerous barriers limiting its potential opportunities. New strategies taking into account the EU’s socio-economic and political environment are deemed necessary.

## Supplementary data

Supplementary data are available at *EURPUB* online.

*Conflicts of interest:* E.-R.P. and M.G. are involved in the European Cancer Organization, closely working towards the implementation of the Europe’s Beating Cancer Plan.

Key points

- Artificial intelligence (AI) has the potential to improve cancer treatment by supporting surgery, clinical imaging and decision-making processes.
- Large disparities are present regarding the geolocation of the clinical trials performed or emerging.
- The current integration of AI for cancer treatment in the European Union (EU) faces numerous socio-economic and regulatory barriers.
- New strategies taking into account EU’s socio-economic and political environment results are crucial.

## Data availability

The data underlying this article are available in the article and in its online supplementary material.

## Supplementary Material

ckac016_Supplementary_Data
