# Supplementary material for: Mapping of clinical research on artificial intelligence in the treatment of cancer and the challenges and opportunities underpinning its integration in the European Union health sector
Source: Eur J Public Health. 2022 Mar 3;32(3):443–9. doi: 10.1093/eurpub/ckac016 (PMC9159319; doi:10.1093/eurpub/ckac016)
Supplement: ckac016_Supplementary_Data [file ckac016_supplementary_data.docx]

SUPPLEMENTARY MATERIAL

*References (cont.)*

1. Jiménez Rodríguez RM, Díaz Pavón JM, de La Portilla de Juan F, Prendes Sillero E, Hisnard Cadet Dussort JM, Padillo J. Prospective randomised study: Robotic- assisted versus conventional laparoscopic surgery in colorectal cancer resection. Cir Esp. 2011;89(7):432–8.
2. Jayne D, Pigazzi A, Marshall H, Croft J, Corrigan N, Copeland J, et al. Effect of robotic-assisted vs conventional laparoscopic surgery on risk of conversion to open laparotomy among patients undergoing resection for rectal cancer the rolarr randomized clinical trial. JAMA - J Am Med Assoc: 2017; 318(16):1569–80.
3. Park IJ, You YN, Schlette E, Nguyen S, Skibber JM, Rodriguez-Bigas MA, et al. Reverse-hybrid robotic mesorectal excision for rectal cancer. Dis Colon Rectum. 2012;55(2):228–33.
4. Colombo PE, Bertrand MM, Alline M, Boulay E, Mourregot A, Carrère S, et al. Robotic Versus Laparoscopic Total Mesorectal Excision (TME) for Sphincter-Saving Surgery: Is There Any Difference in the Transanal TME Rectal Approach?: A Single-Center Series of 120 Consecutive Patients. Ann Surg Oncol. 2016; 1:23(5):1594–600.
5. van Loon JWL, Smeele LE, Hilgers FJM, van den Brekel MWM. Outcome of transoral robotic surgery for stage I–II oropharyngeal cancer. Eur Arch Oto-Rhino- Laryngology. 2015;272(1):175–83.
6. Gupta NP, Nayyar R, Singh P, Anand A. Robot-assisted adrenal-sparing surgery for pheochromocytoma: Initial experience. J Endourol. 2010 Jun 1;24(6):981–5.
7. Phee SJ, Reddy N, Chiu PWY, Rebala P, Rao G V., Wang Z, et al. Robot-Assisted Endoscopic Submucosal Dissection Is Effective in Treating Patients With Early- Stage Gastric Neoplasia. Clin Gastroenterol Hepatol. 2012;10(10):1117–21.
8. Weizer AZ, Palella G V., Montgomery JS, Miller DC, Hafez KS. Robot-assisted retroperitoneal partial nephrectomy: Technique and perioperative results. J Endourol. 2011 Apr 1;25(4):553–7.
9. van der Sluis PC, Ruurda JP, van der Horst S, Verhage RJJ, Besselink MGH, Prins MJD, et al. Robot-assisted minimally invasive thoraco-laparoscopic esophagectomy versus open transthoracic esophagectomy for resectable esophageal cancer, a randomized controlled trial (ROBOT trial). Trials. 2012 Nov 30;13.
10. Ko BS, Wang YF, Li JL, Li CC, Weng PF, Hsu SC, et al. Clinically validated machine learning algorithm for detecting residual diseases with multicolor flow cytometry analysis in acute myeloid leukemia and myelodysplastic syndrome. EBioMedicine. 2018 Nov 1;37:91–100.
11. Skrede OJ, De Raedt S, Kleppe A, Hveem TS, Liestøl K, Maddison J, et al. Deep learning for prediction of colorectal cancer outcome: a discovery and validation study. Lancet. 2020 1;395(10221):350–60.
12. Wagner S, Vadakekolathu J, Tasian SK, Altmann H, Bornhäuser M, Pockley AG, et al. A parsimonious 3-gene signature predicts clinical outcomes in an acute myeloid leukemia multicohort study. Blood Adv. 2019;3(8):1330–46.
13. Dai JY, LeBlanc M, Goodman PJ, Scott Lucia M, Thompson IM, Tangen CM. Case- only methods identified genetic loci predicting a subgroup of men with reduced risk of high-grade prostate cancer by finasteride. Cancer Prev Res. 2019 Feb 1;12(2):113–20.
14. Zhong Q, Fang J, Huang Z, Yang Y, Lian M, Liu H, et al. A response prediction model for taxane, cisplatin, and 5-fluorouracil chemotherapy in hypopharyngeal carcinoma. Sci Rep. 2018 Dec 1;8(1).
15. Wan XB, Zhao Y, Fan XJ, Cai HM, Zhang Y, Chen MY, et al. Molecular prognostic prediction for locally advanced nasopharyngeal carcinoma by support vector machine integrated approach. PLoS One. 2012 Mar 9;7(3).
16. Niikura R. Artificial Intelligence Versus Expert Endoscopists for Diagnosis of Gastric Cancer - ClinicalTrials.gov: https://clinicaltrials.gov/ct2/show/NCT04040374 . Forthcoming. Accessed 13 June 2020.
17. Zui X. Development and Validation of a Deep Learning Algorithm for Bowel Preparation Quality Scoring - ClinicalTrials.gov. <https://clinicaltrials.gov/ct2/show/NCT03908645>. Forthcoming. Accessed 13 June 2020.
18. Kurnaz S, Johnston K. SYNERGY-AI: Artificial Intelligence Based Precision Oncology Clinical Trial Matching and Registry- ClinicalTrials.gov https://clinicaltrials.gov/ct2/show/NCT03452774?term=%28artificial+intelligence +OR+machine+learning+OR+deep+learning+OR+neural+network%29+AND+% 8treatment+OR+therapy%29+AND+%28effectiveness+OR+potential%29&cond= cancer+OR+tumour*+OR+malignan*&draw=2. Forthcoming. Accessed 13 June 2020.
19. Honggang Y. A Single Center Study on the Effectiveness and Safety of Polyp Classification With Artificial Intelligence- ClinicalTrials.gov: https://clinicaltrials.gov/ct2/show/NCT04216901?term=%28artificial+intelligence +OR+machine+learning+OR+deep+learning+OR+neural+network%29+AND+%2 8treatment+OR+therapy%29+AND+%28effectiveness+OR+potential%29&cond= cancer+OR+tumour*+OR+malignan*&draw=2&rank=1. Forthcoming. Accessed 13 June 2020.
20. Chen P-J. Computer-aided Detection for Colonoscopy - ClinicalTrials.gov: <https://clinicaltrials.gov/ct2/show/NCT03842059>. Forthcoming. Accessed 12 June 2020.
21. EIT Health, McKinsey & Comapany. Transforming healthcare with AI. 2020.
22. Delponte L. European Artificial Intelligence (AI) leadership, the path for an integrated vision. 2018.
23. Gómez-González E, Gómez E. Artificial Intelligence in Medicine and Healthcare: applications, availability and societal impact: https://publications.jrc.ec.europa.eu/repository/bitstream/JRC120214/jrc120214_ ai_in_medicine_and_healthcare_report-aiwatch_v50.pdf. Accessed 2 July 2020.
24. Watson W, Marsh C. Artificial Intelligence Bias in Healthcare: https://www.boozallen.com/c/insight/blog/ai- bias-in-healthcare.html. Accessed 2 July 2020.
25. Spatharous A, Hieronimus S, Jenkins J. Transforming healthcare with AI: The impact on the workforce and organizations | McKinsey: https://www.mckinsey.com/industries/healthcare- systems-and-services/our-insights/transforming-healthcare-with-ai# . Accessed 2 July 2020.
26. Davenport T, Kalakota R. The potential for artificial intelligence in healthcare. Futur Healthc J: /pmc/articles/PMC6616181/?report=abstract. Accessed 2 July 2020.
27. Prenuvo. Why late diagnosis of cancer and disease: https://www.prenuvo.com/customer/why-we-detect- disease-late. Accessed 2 July 2020.
28. World Cancer Research Fund. Worldwide cancer data: <https://www.wcrf.org/dietandcancer/worldwide-cancer-data/>. Accessed 22 November 2021.
29. OECD. Health at a Glance: Europe 2018 STATE OF HEALTH IN THE EU CYCLE. <https://doi.org/10.1787/health_glance_eur-2018-en>. Accessed 2 July 2020.
30. Breens B. INNOVATION FUNDING TRENDS FOR PERSONALIZED MEDICINE. 2017.
31. Eur-Lex. Annexes to the Communication from the Commission to the European Parliament and the Council Europe’s Beating Cancer Plan: https://eurlex.europa.eu/resource.html?uri=cellar:8dec84ce-66df-11eb-aeb5- 01aa75ed71a1.0002.02/DOC_2&format=PDF2021. Accessed 11 July 2021.

*Table A1.* Europe’s Beating Cancer Plan List of action. Adapted from *Annexes to the Communication from the Commission to the European Parliament and the Council Europe’s Beating Cancer Plan*^71^

| Action | Timeline |
| --- | --- |
| A modern approach to cancer: new technologies, research and innovation at the service of patient-centred cancer prevention and care | |
| Create a Knowledge Centre on Cancer to facilitate the coordination of scientific and technical cancer-related initiatives at EU level | 2021 |
| Launch a European Cancer Imaging Initiative to support the development of new computer aided tools to improve personalised medicines and innovative solutions | 2022 |
| (i) Enable cancer patients to securely access and share electronic health records for prevention and treatment across borders through the European Health Data Space  (ii) Develop a repository of digital twins in healthcare, including for more individual cancer treatment  (iii) Expand the European Cancer Information System  (iv) Launch Horizon Europe partnerships: the Innovative Health and Care Systems | 2021-2025 |
| Improving early detection of cancer |  |
| Cancer Imaging Initiative to support the development of new computer-aided tools to improve personalised medicine and innovative solutions | 2021-2025 |
| Ensuring high standards in cancer care |  |
| (i) Creation of National Comprehensive Cancer Centre(s) in all Member States and EU network by 2025  (ii) EU cancer Treatment Capacity and Capability Digital Mapping project | 2021-2025 |
| “Cancer Diagnostic and Treatment for All” Initiative to improve access to innovative cancer diagnosis and treatments | 2021-2025 |
| Launch the European Initiative to understand Cancer (UNCAN.eu) | 2021-2025 |
| Create an EU platform to improve access to cancer medicines to support the repurposing of existing molecules with a harmonized and sustainable EU dimension | 2021-2025 |
| Implementation of the legal framework for clinical trials | 2021-2025 |
| Adoption of the Regulation on Health Technology Assessment | 2021 |
| (i) Ser up Partnership on Personalised Medicine to identify priorities for research and education in personalised medicine, support research projects relevant to cancer prevention, diagnosis and treatment  (ii) Development of a roadmap to personalised prevention | 2023-2025 |
| Launch the ‘Genomic for Public Health’ project along with the 1+ Million Genomes Initiative, to ensure the access to large amounts of genomic data for research, prevention and personalised medicine | 2021-2025 |
| (i) Launch a new project using High-Performance Computing to rapidly test existing molecules and new drug combinations (ii) Support collaborative projects on cancer diagnostics and treatment using High-Performance Computing and AI (iii) Assist researchers working on personalised cancer treatments through tailored support and new digital platforms | 2021-2027 |
| Improving the quality of life for cancer patients, survivors, and careers |  |
| Flagship: ‘Better life for cancer patients’ initiative | 2021-2023 |
| Reducing cancer inequalities across the EU |  |
| Flagship: Establish a Cancer Inequalities Registry to map inequalities between Member States and regions | 2021-2022 |
| (i) Strengthen e-health, telemedicine, and remote monitoring systems  (ii) promote the virtual consultation model of the ERNs | 2021-2023 |

*Table B1*. Extended synthesis of studies included in the systematic literature review

| **Articles on AI applicability in the treatment of cancer focusing on clinical imaging techniques** | | | | | | |
| --- | --- | --- | --- | --- | --- | --- |
| **Ref** | **Author(s), year, location, status** | **Title** | **Population** | **Intervention** | | **Outcomes** |
| [17] | Sayesteh et. al; 2019; Iran; study completed | Neo-adjuvant chemoradiotherapy response prediction using MRI based ensemble learning method in rectal cancer patients | 98 patients with rectal cancer divided into a training (n=53) and a  validation set (n=45) | All patients underwent MRI a week before the neo-adjuvant chemoradiotherapy. Several features were, extracted from the MR images and SVM Bayesian network, neural network and KNN classifiers were used individually and together for response prediction. Predictive performance was evaluated using the area under the receiver operator characteristic (ROC) curve (AUC). | | The best result was for the Bayesian network classifier in the non-pre-processed MR image, with an AUC and accuracy of 75.2 percent and 80.9 percent, respectively, which was validated in the validation set with AUC and accuracy of 74 percent and 79 percent. In EMLMs, the best result was for a four-classifier EMLM (SVM.NN.BN.KNN) with AUC and accuracy of 97.8% and 92.8 percent in testing and 95 percent and 90 percent in validation sets, respectively.  Conclusion: In patients with rectal cancer, machine learning algorithms can be utilized to predict nCRT response. |
| [18] | de Jong et. al; 2018; multicentric clinical trial; study completed | Applicability of Prognostic CT-based  Radiomic Signature  Model Trained on  Stage I-III Non-Small  Cell Lung Cancer in  Stage IV Non-Small  Cell Lung Cancer | Two datasets of chemo-naive stage IV adenocarcinoma patients were investigated (n=285; monocentric and n=223; multicentric). 195 patients eligible | For the patients' main tumour, radiomic characteristics were calculated. To see if radiomic features of CT scans provide predictive information, the c-index of cox regression was generated and compared to the signature performance for overall survival. | | Patients with a prognosis index below the signature median (=92) had a significantly better overall survival than those with a prognostic index above the median.  Conclusion: For stage IV NSCLC, a radiomic signature obtained from daily practice CT images has predictive relevance. |
| [19] | Xiong et. al; 2018; China; completed | The Role of PET-Based Radiomic Features in Predicting Local Control of Oesophageal Cancer Treated with Concurrent Chemoradiotherapy | 30 patients suffering from oesophageal squamous cell carcinoma, previously enrolled in a CT (NCT01843049) and treated with concurrent CRT between April 2021 and June 2015 | Before CRT (pre-CRT) and after 20 fractions of radiation doses, all patients had PET/CT scans (mid-CRT). All patients received definitive concurrent CRT, and follow-up evaluations were performed one month after all treatments were completed, then every three months for the next two years, and then every six months thereafter. On pre-CRT PET imaging, a tumour volume of interest was determined for each patient. 440 radiomic characteristics were collected from both pre-CRT and mid-CRT PET scans for each of the 30 patients recruited. As discriminative characteristics, the top 25 features with the highest areas under the receiver operating characteristic curve for determining local control status were chosen. Random forest (RF), support vector machine (SVM), logistic regression (LR), and extreme learning machine (ELM) were used to develop predictive models containing clinical, radiomic, or a combination of both clinical and radiomic variables. | | With an accuracy of 93.3 percent, a specificity of 95.7 percent, and a sensitivity of 85.7 percent, an RF model including both clinical and radiomic variables had the best predictive performance. The 2-year local control rate and PFS rate in the low-risk group were 100.0 percent (95 percent CI 100.0–100.0 percent) and 52.2 percent (31.8–72.6 percent), respectively, and 14.3 percent (0.0–40.2 percent) and 0.0 percent (0.0–40.2 percent) in the high-risk group, based on risk scores of local failures predicted by this model.  Conclusion: This model can stratify patients with varied chances of local failure following CRT for oesophageal cancer, making individualized treatment more efficient. |
| [20] | Zhao et al; 2010; USA; Completed | A Support Vector Machine (SVM) for Predicting Preferred Treatment Position in Radiotherapy of Patients With Breast Cancer | 400 patients with breast cancer (n=200; left-breast and n=200; right-breast) | The organs at danger and the tumour bed's three-dimensional geometric features were retrieved. Patients were classified into the prone or supine classes using a two-stage classifier. The authors separate the patients into two groups depending on their in-field heart volume in the first stage using basic thresholding. The prone position was identified as the preferred treatment posture for individuals with an in-field heart volume of less than 0.1 cc. A weighted support vector machine will be used to further classify patients with in-field heart volumes greater than 0.1 cc in the second stage (SVM). The SVM's weight settings were tweaked to increase specificity while lowering sensitivity while keeping a fair level of sensitivity. The authors performed k-fold cross validations to evaluate the SVM classifier's performance. A feature selection technique was also employed to find the best-performing features for classification. | | The proposed method's overall sensitivity and specificity were found to be 90.4 percent and 99.3 percent, respectively. When the authors used two-stage categorization, the proportion of prone-treated patients who needed a second supine CT scan dropped to 16.3/170, or 9.6%, compared to 21/170, or 12.4%, when they just used the first stage (thresholding).  Conclusion: Based on features derived from prone CT scans, the authors' work demonstrated that a feature-based classifier can identify the preferred treatment position. The two-stage classifier produced a high level of specificity while sacrificing sensitivity. |
| [21] | Chen et al; 2013; USA; completed | Objected constrained registration and manifold learning: A new patient setup approach in image guided radiation therapy of thoracic cancer | 11 lung cancer patients | Before the first treatment, all patients had a long training fluoroscopy, and many short testing fluoroscopies were obtained weekly during the preparatory patient setup of image guided radiation (IGRT). Three phases were involved in the data analysis: first, a 4D target motion model was created using 4DCT and projected to the training fluoroscopy using deformable registration. The target motion (kinetic) and position (static) information in the training fluoroscopy were then used to generate a 2D subspace using the manifold learning method. Following that, the breathing phase in the fluoroscopy examination was determined by locating it in the subspace. Finally, the pre-treatment patient position adjustment for the IGRT was calculated by registering the phase determined testing fluoroscopy to the matching 4DCT. Clinical picture sets and numerical phantoms were used to test the approach. | | With over 98 percent volume similarity in 4DCT and over 95 percent area similarity in training fluoroscopy, the registration effectively recreated the 4D motion model. With less than 3% phase inaccuracy, the machine learning algorithm determined phase values in over 98 percent and 93 percent of test photos of the phantom and patient, respectively.  Conclusion: The suggested approach has the potential to increase the accuracy of pre-treatment setup for patients with thoracic cancer by integrating registration and machine learning. |
| *  [22] | Zhao et al; estimated 2024; China; Not yet completed | The AI Prognostic Assessment and Pathological Basis Research of Early HCC After Minimally Invasive Treatment | Estimated 1200 patients with hepatocellular carcinoma | All the patients with hepatocellular carcinoma (HCC) received minimally invasive treatment, such as transcatheter arterial chemoembolization (TACE), radiofrequency ablation (RFA), or a combination of the two. The goal of this study is to predict patient outcomes after minimally invasive treatment is completed. | |  |
| *  [23] | Whan et al., estimated 2021; China; Not yet completed | Post-Neoadjuvant Treatment MRI Based AI System to Predict pCR for Rectal Cancer | Estimated 322 patients with rectal cancer | Patients with locally advanced rectal cancer (LARC) who have completed neoadjuvant treatment but have not yet had total mesorectum excision (TME) surgery will be included in the study. The artificial intelligence system will collect the features of each enrolled patient's post-neoadjuvant therapy MRI scans, which will then be assessed by qualified radiologists. Both techniques, which are blind to the pathologic report of the TME material, produce a predicted pathologic response to neoadjuvant treatment for each enrolled patient, which is shown as pCR or non-pCR. | |  |
| *  [24] | Juneau et al., estimated 2024; Canada; not yet completed | PSMA-PET: Deep Radiomic Biomarkers of Progression and Response Prediction in Prostate Cancer | Estimated 1000 patients with prostate cancer | PET and PSMA scans will be used to collect images from all patients. The goal of this research is to create a predictive imaging biomarker that can be used to predict treatment outcomes. | |  |
| *  [25] | Zhang et al; estimated 2025; China; not yet completed | Prediction of Therapeutic Response of Apatinib in Recurrent Gliomas | Estimated 600 cancer patients presenting gliomas | All patients with recurrent gliomas who are treated with Apatinib will have their images taken. The purpose of this study is to assess Apatinib's efficacy and safety. Furthermore, this study aims to build and optimize MR and histopathological imaging-based algorithms that can predict Apatinib responses in patients with recurrent gliomas using artificial intelligence. The development of a registry for patients with recurrent gliomas treated with Apatinib that includes detailed survival data, radiological data, and histopathological imaging data, as well as a large enough sample size for artificial intelligence, allows for tailored prediction of Apatinib responses. | |  |
| *  [26] | Xin et al; estimated 2022; China; not yet completed | Establishment and Evaluation of Multimodal Image Recognition System of Glioma Based on Deep Learning | Estimated 350 cancer patients presenting gliomas | The project team uses non-invasive molecular imaging technology to define the characteristics of molecular subsets of glioblastoma based on tumour metabolic data, based on the construction of a glioma molecular type system. It has great potential in clinical research of glioma diagnosis, prognosis, and treatment options by combining deep learning-based target detection and image recognition with big data analysis, which could provide a scientific basis for the establishment and promotion of glioma molecular analysis and recognition system. | |  |
| *  [27] | Xu et al; estimated 2022; China; not yet completed | Study on Adaptive Radiotherapy and Multimodal Information of Cervical Cancer Assist ed by Artificial Intelligence (SOARAM IOCC) | Estimated 122 cervical cancer patients in a randomised clinical trial | Patients in the experimental group received concurrent adaptive external volumetric rotating intensity modulated radiation and chemotherapy, followed by image-guided adaptive brachytherapy. After 15 fractions of external radiotherapy, CT repositioning will be done, after which a new target volume will be contoured, and a new radiotherapy plan will be produced with the help of an artificial intelligence program. From the 17th fraction external radiotherapy, a new treatment strategy will be implemented. The researchers will gather data on side effects, survival, dosimetry, imaging, clinical characteristics, and cost-effectiveness. The statistical analysis is as follows. First is the difference in grade 3 side effects between the two groups. Second is 2-year PFS and OS differences between the two groups. Third is relationship between dosimetric differences and prognosis. Fourth one is to analyse the prognostic and predictive factors of adaptive radiotherapy from the patient's clinical characteristics, Positron emission tomography-computed tomography (PET/CT), Magnetic Resonance Imaging (MRI) and other multimodal information. Fifth is cost-benefit analysis of Artificial Intelligence (AI). | |  |
| *  [28] | Wing et al; estimated 2024; China; not yet completed | Artificial Intelligence in Mammography-Based Breast Cancer Screening | Estimated 1000 breast cancer patients | The purpose of this proposed study is to investigate the breast cancer diagnostic performance of the AI-CAD system that is used to read mammograms. To identify and describe worrisome breast lesions on mammograms, we will use a commercially available AI-CAD product based on deep-learning algorithms (IBM Watson Imaging AI Solution). The prospective cancer lesions can be tagged, and their mammographic characteristics and likelihood of malignancy will be recorded automatically. Following the AI post-processing, we will conduct statistical analysis to establish the accuracy of the AI-CAD system in predicting BC risk. | |  |
| *  [29] | Mutter et al; estimated 2021; France; not yet completed | Endoscopic Optical Imaging for Precision Oncology Treatment Applied to Colorectal Tumours (Elios-Color-on-Specimen) | Estimated 34 colorectal cancer patients | FF-OCT (Light-CT Scanner, LLTechSAS, Paris, France) is another cutting-edge optical imaging technique accessible at the IHU, allowing for non-destructive and high-resolution optical biopsy without the need for tissue treatment.  The working premise is that, when compared to immunohistochemistry, molecular fluorescence enhanced reality provides for more precision in the distinction of tumour tissue and healthy tissue in patients with colorectal cancer.  This technology will be compared to hyperspectral imaging (HSI TIVITA system) and optical imaging (FF-OCT system), two potentially useful methods for tumour tissue detection. | |  |
|  |  |  | **Articles on AI applicability in the treatment of cancer focusing on robotic surgery** | |  |  |
| **Ref** | **Author(s), year, location, status** | **Title** | **Population** | **Intervention** | | **Outcomes** |
| [30] | Asimakapolous et al; 2011; USA; completed | Randomized  Comparison Between  Laparoscopic and  Robot-Assisted Nerve Sparing Radical  Prostatectomy | 128 male patients were randomized in two groups and treated by a single experienced surgeon with traditional LRP (Group I-64 patients) or RALRP (Group II-64 patients) | Between 2007 and 2008, 128 male patients were randomly assigned to one of two groups and treated by a single experienced surgeon with either classic LRP (Group I-64 patients) or RALRP (Group II-64 patients), with the goal of bilateral interfacial nerve sparing in all cases. The primary goal was to compare 12-month erectile function (EF) results. Oncological outcomes, complication rates, and continence outcomes were also compared. | | Operating time, anticipated blood loss, transfusion rate, complications, rates of positive surgical margins, rates of biochemical recurrence, continence, and time to continence all showed no statistically significant differences. However, a 12-month assessment of intercourse capability (with or without phosphodiesterase type 5 inhibitors) revealed that RALRP had a clear and significant advantage (32 percent vs. 77 percent, P 0.0001). For intercourse, RALRP had a significantly shorter time to capability. RALRP had significantly greater rates of return to baseline International Index of Erectile Function (IIEF-6) EF domain score questionnaires (questions 1–5 and 15) (25 percent vs. 58 percent) and IIEF-6 > 17 (38 percent vs. 63 percent) (P = 0.0002 and P = 0.008, respectively).  Conclusion: Our study provides the first high-level proof that RALRP improves EF recovery much more than LRP without compromising the procedure's oncologic radicality. Larger RCTs are required to determine whether a new gold-standard treatment for RP has emerged. |
| [31] | Engel et al; 2011; USA (multicentre);completed | Changes in Penile Length After Robot Assisted Laparoscopic Radical Prostatectomy | 127 males with prostate cancer; 94 completed the 11-month follow-up | Men with normal erectile function who undergone bilateral nerve-sparing radical prostatectomy were enrolled in a randomized, open-label, multicentre research. A selection of men from a single site who received RALRP by one surgeon were analysed for changes in measured stretched penile length (SPL), a secondary end aim of the study. For 9 months, they were given either intraurethral alprostadil 125 to 250 mg daily or oral sildenafil citrate 50 mg daily. SPL was measured with a semirigid ruler from the pubic bone to the coronal sulcus before surgery and at 1, 3, 6, 9, 10, and 11 months. | | The average age of the patients was 56.5 years. Before surgery, the mean SPL (cm) was 11.77, and after one month, it was 11.13 (P 0.0001). At 3 and 6 months, there was a trend toward SPL recovery. At 9, 10, and 11 months, mean SPL did not differ substantially from baseline.  Conclusion: The alterations in SPL after RALRP for prostate cancer are described in this article. Shortly after surgery, the predicted drop in length was noticed, however by 9 months, penile length had restored to the preoperative measurement. |
| [32] | Pierro et al; 2011; Switzerland; completed | A prospective trial comparing consecutive series of open retropubic and robot- assisted laparoscopic radical prostatectomy in a centre with a limited caseload | 150 patients with prostate cancer involved divided in two equal comparison groups (n=75) | RRP was performed on 75 patients in a row, and RALP was performed on 75 patients in a row, encompassing all patients on the learning curve. The baseline characteristics of the patients, as well as their perioperative and postoperative outcomes and complications, were assessed. At 3- and 12-month follow-up, the researchers looked at oncologic data (positive margins, PSA), perioperative complications, urine continence, and erectile function. | | The positive surgical margin (PSM) rates for RRP and RALP were 32 percent and 16 percent, respectively (p=0.002). The PSA value was 0.2 ng/ml in 91 percent and 88 percent of RRP and RALP patients 3 months after surgery (p=0.708) and 87 percent and 89 percent of patients 12 months after surgery (p=0.36), respectively. RRP and RALP continuity rates were 83 percent and 95 percent at 3-month follow-up (p=0.003), respectively, and 80 percent and 89 percent at 12-month follow-up (p=0.092). Before RRP and RALP, patients who were potent without phosphodiesterase type 5 inhibitors (PDE5-I) were found to be more potent. Erectile function was restored in 25% (12 of 49 patients) and 68 percent (25 of 37 patients) 3 months after surgery (p=0.009), and in 26 percent (12 of 47 patients) and 55 percent (12 of 22 patients) 12 months after surgery (p=0.009), respectively. The RRP group had a 12-month minimum follow-up, while the RALP group had a 12-month median follow-up (range: 3-12). Major complication rates for RRP and RALP were 28 percent and 7 percent (p=0.025), respectively, according to the modified Clavien system; mild complication rates were 24 percent and 35 percent (p=0.744), respectively.  Conclusion: Despite having a smaller caseload and considering the learning curve, RALP outperforms RRP in terms of PSM and severe complications, urine continence and erectile function. |
| [33] | Porpiglia et al; 2013; Italy; completed | Randomized controlled trial comparing laparoscopic and robot-assisted radical prostatectomy | 120 prostate cancer patients involved in a randomized clinical trial divided in two equal comparison groups (n=60) | Between January 2010 and January 2011, 120 patients with organ-confined prostate cancer were enrolled and were assigned to one of two groups based on surgical approach: the RARP group or the LRP group (using a randomization strategy). All RARP and LRP procedures were carried out by the same surgeon using the same approach. The demographic, perioperative, and pathologic outcomes, including complications and PSA readings, were all documented and compared. Continence and potency were assessed at the moment of catheter removal and 48 hours later, as well as after 1, 3, 6, and 12 months. Statistics included the student t test, Mann-Whitney test, (2) test, Pearson (2) test, and multiple regression analysis. | | In terms of perioperative and pathologic outcomes, complication rate, and PSA levels, no changes were found. At every time point, the RARP group had a greater continence rate: after three months, the RARP group had an 80 percent continence rate and the LRP group had a 61.6 percent continence rate (p=0.044), and after one year, the continence rates were 95.0 percent and 83.3 percent, respectively (p=0.042). The percentage of erection recovery was 80.0 percent and 54.2 percent, respectively, among preoperatively potent patients treated with nerve-sparing procedures (p=0.020). The minimal number of patients was one of the drawbacks.  Conclusion: RARP produced improved functional outcomes in terms of continence and potency recovery. More research is needed to corroborate our findings. |
| [34] | Tan et al; 2016; USA; completed | Technique and outcomes of bladder neck intussusception during robot-assisted laparoscopic prostatectomy: A parallel comparative trial | 48 prostate cancer patients involved divided in two equal groups (n=24) | A study of 48 males who had a robot-assisted laparoscopic prostatectomy with bladder neck intussusception (n = 24) and no intussusception (n = 24) was conducted. 3-0 polyglycolic acid horizontal mattress sutures were used anterior and posterior to the bladder neck to close the intussusception. We looked at baseline parameters as well as clinicopathologic results. We used the urine domain of the Expanded Prostate Cancer Index-Short Form to compare urinary function at 2 days, 2 weeks, 2 months, and last follow-up after adjusting for age, BMI, race, and DAmico risk classification. | | Between treatment groups, baseline patient characteristics and clinicopathologic outcomes were similar (P>0.05). The incidence of serious postoperative complications (4.2 percent vs. 4.2 percent, P = 1.000) and the median catheter duration (8 vs. 8d, P = 0.125) were not different. In adjusted analyses, urine scores for the intussusception arm were substantially higher at 2 weeks (65.4 vs. 46.6, P = 0.019) before converging at 2 months (69.1 vs. 68.3, P = 0.929) after catheter removal and at the final follow-up (median = 7mo, 80.5 vs. 77.0; P = 0.665).  Conclusion: Bladder neck intussusception is possible and safe during robot-assisted laparoscopic prostatectomy. Although the long-term consequences of intussusception appear to be limited, it may enhance urine function during the early stages of recovery. |
| [35] | Chang et al; 2018; Korea; completed | Retzius-sparing robot assisted radical prostatectomy using the Revo-i robotic surgical system: surgical technique and results of the first human trial | 17 patients with prostate cancer | Patients were treated with the Revo-i at our urology department between August 17, 2016, and February 23, 2017. Retzius-sparing robot-assisted radical prostatectomy was performed on the patients (RS-RARP). The major goal was to describe the RS-RARP surgical procedure in detail utilizing the Revo-i. Additionally, the Revo-safety i's was evaluated based on intra-operative and postoperative problems within 30 days of surgery. Surgical margin status and biochemical recurrence were also used to assess early oncological outcomes (BCR). The use of no or only one pad was characterized as continence. The satisfaction of surgeons with the Revo-i was measured using a Likert scale. | | 8 minutes, 92 minutes, 26 minutes, and 200 mL were the median docking time, console time, ureterovesical anastomosis time, and estimated blood loss, respectively. A blood loss of 1500 mL necessitated intra-operative transfusion for one patient. Two patients required blood transfusions after surgery, but there were no other critical or major problems. The average length of stay in the hospital was four days. Four patients had positive surgical margins at three months, one had BCR, and 15 were continent. The Revo-i performed well for most surgeons.  Conclusion: The Revo-i robotic surgical system was successfully used to treat patients with localized PCa in the first human research. The perioperative, early oncological, and continence results are all positive. More prospective studies are needed to back up our early findings. |
| [36] | Shah & Abazza; 2011; USA; completed | Comparison of Intraoperative Outcomes Using the New and Old Generation Da Vinci® Robot for Robot Assisted Laparoscopic Prostatectomy | 100 consecutive robotic prostatectomy cases were revised and compared | The researchers examined intraoperative results for procedures conducted on either the da Vinci S robot or a first-generation standard robot in 100 consecutive robotic prostatectomy patients. Between the two groups, baseline demographic data and intra-operative variables that could affect results were evaluated and compared. | | The average overall operative time with the conventional da Vinci robot was 191 minutes (range 132-266), compared to 169 minutes with the S robot (range 98-230), a difference of 22 minutes (P = 0.002). Despite no difference in mean patient BMI of 30.6 (range 19-51) for standard versus 29.3 (range 21-37) for S (P = 0.31), no difference in mean prostate size of 54.6 g (range 26-101) for standard versus 57.3 g (range 32-151) for S (P = 0.55), and no difference in nerve-sparing frequency (P = 0.99), this difference was statistically significant.  There was no difference in the proportions of procedures conducted by residents, which ranged from none to the full process in certain cases, but the standard was employed more frequently for the surgeon's first case of the day (P = 0.006). Blood loss (P = 0.08), positive margins (P = 0.87), and the mean number of lymph nodes excised were all the same (10.7 vs 10.6).  Conclusion: Both generations of da Vinci robotic technology are equally successful for PALP, however the S robot looks to take less time. As incremental breakthroughs continue, more such reviews are required to inform institutions and public policy decision-makers on investments in newer generations of robotic technology. |
| [37] | Chung et al; 2011; Korea; completed | Comparison of Oncological Results, Functional Outcomes, and Complications for Transperitoneal Versus Extraperitoneal Robot-Assisted Radical Prostatectomy: A Single Surgeon's Experience | 265 patients with prostate cancer involved in a randomized clinical trial | 105 patients had TP robotic radical prostatectomy and 155 patients had EP robotic radical prostatectomy between June 2007 and April 2009. The two groups' clinicopathological and perioperative data were compared. Potency and incontinence were measured as well as postoperative complications and functional results. | | Positive surgical margins did not differ significantly across the groups. There were no significant differences in total operational time, number of lymph nodes excised, or predicted blood loss. However, the EP group spent less time on the robot console than the TP group (89.1 vs. 107.8 minutes, p = 0.03). The EP group had lower postoperative pain scale scores than the TP group (2.7 vs. 6.3, p 0.001). The EP group had a decreased frequency of ileus and hernia, but a higher incidence of lymphocele. The rates of postoperative potency and continence were equal in both groups; however, the EP group recovered continence faster than the TP group.  Conclusion: The EP technique had similar oncological and perioperative outcomes to the TP strategy, as well as less postoperative discomfort, fewer bowel-related complications, and superior functional outcomes. In robotic radical prostatectomy, the EP method could be a viable option. |
| [38] | Nix et al; 2010; USA; completed | Prospective Randomized Controlled Trial of Robotic versus Open Radical Cystectomy for Bladder Cancer: Perioperative and Pathologic Results | 41 bladder cancer patients were included for a randomized clinical trial | Twenty-one of the 41 patients who had surgery were randomly assigned to the robotic approach and the other twenty to the open procedure. Radical cystectomy, bilateral pelvic lymphadenectomy, and urine diversion using an open or robotic-assisted laparoscopic method. The lymph node (LN) yield was the key end point, with a noninferiority margin of four LNs. Demographic variables, perioperative outcomes, pathologic results, and short-term narcotic use were all secondary end goals. | | There were no significant variations in age, sex, BMI, American Society of Anaesthesiologists categorization, aspirin anticoagulation regimen, clinical stage, or diversion type between the two groups. Operating room time, estimated blood loss, time to flatus, time to bowel movement, and use of inpatient morphine sulphate equivalents all showed significant disparities. In terms of overall complication rate or hospital stay, there was no substantial difference. On surgical pathology, 14 patients in the robotic group had pT2 or higher cancer, 3 patients had pT3/T4 cancer, and 4 patients had node-positive cancer. Eight patients in the open group had pT2 or higher disease, five had pT3/T4 disease, and seven had node-positive disease. In the robotic group, 19 LNs were removed on average, compared to 18 in the open group.  Conclusion: We describe the findings of a prospective randomized controlled noninferiority research with an LN yield as the primary end point, revealing that the robotic approach is noninferior to the open approach. In several perioperative characteristics, the robotic technique outperforms the open approach. |
| [39] | Richards et al; 2011; USA; completed | Does initial learning curve compromise outcomes for robot-assisted radical cystectomy? A critical evaluation of the first 60 cases while establishing a robotics program. | 60 bladder cancer patients were involved | From January 2008 to March 2010, 60 patients with clinically localized bladder cancer underwent RARC with PLND. Analysis of variance was used to analyse the effect of LC on the patients, who were divided into tertiles. | | The demographics and clinical characteristics of patients in each tertile were similar. The mean total operative time decreased from the first to third tertiles, from 525 to 449 minutes (P=0.059). The average estimated blood loss remained constant across tertiles. The number of complications reduced as the LC progressed, from 14 (70%) in the first tertile to 6 (30%) in each of the second and third tertiles (P0.013). Across tertiles, the mean total lymph node yield and number of positive margins remained unchanged.  Conclusion: For surgeons who are skilled in ORC, RARC with PLND can be performed safely in a high-volume, recently established robotic surgery program with an experienced team without compromising operational, postoperative, or short-term pathologic outcomes during the LC. |
| [40] | Parekh et al; 2013; USA; completed | Perioperative outcomes and oncologic efficacy from a pilot prospective randomized clinical trial of open versus robotic assisted radical cystectomy | 47 bladder cancer patients involved in a randomised clinical trial (data available on 40 patients for analysis) | From July 2009 to June 2011, a pilot prospective randomized trial comparing the perioperative outcomes and oncologic efficacy of open vs robotic aided laparoscopic radical cystectomy for consecutive patients was conducted. | | Oncologic outcomes of positive margins (5 percent each, p = 0.50) or number of lymph nodes removed for open radical cystectomy (23, IQR 15-28) vs robotic aided laparoscopic radical cystectomy (11, IQR 8.75-21.5) groups (p = 0.135) were not significantly different. In comparison to the open radical cystectomy group (800 ml, IQR 400-1,100), the robotic assisted laparoscopic radical cystectomy group (400 ml, IQR 300-762.5) had lower estimated blood loss and a lower rate of excessive length of stay (more than 5 days) (65 percent vs 90 percent, p = 0.11). In addition, the robotic group required fewer transfusions (40 percent versus 50 percent, p = 0.26).  Conclusion: The concept of randomly assigning patients with bladder cancer undergoing radical cystectomy to an open or robotic technique is supported by our research. Our findings indicate that surrogates of oncologic efficacy do not differ significantly. When compared to open radical cystectomy, robotic assisted laparoscopic radical cystectomy has the potential to reduce projected blood loss and hospital stay. A bigger multicentre prospective randomized clinical trial is needed to confirm our findings. |
| [41] | Jiménez et al; 2011; Spain; completed | Prospective randomised study: Robotic- assisted versus conventional laparoscopic surgery in colorectal cancer resection | 56 colorectal cancer patients were involved in a randomized clinical trial | Between January 2008 and January 2009, 56 patients with colorectal cancer were randomly assigned to either the robotic or laparoscopic groups. The researchers compared age, BMI, tumour location, conversions in each group, problems during and after surgery, and the histological characteristics of the tissues acquired. | | There were no statistically significant differences in age (P=.055), BMI (P=.12), or tumour site (P=.91). Only one patient in the robotic group and none in the laparoscopic group required a transfusion. The percentage of conversions was the same in both groups, but the preparation and operating times in patients who used the robotic device were significantly longer (P=.0001 and P=.017, respectively). In terms of the rate of complications or the percentage of re-interventions, there were no changes (14.2 percent and 7.1 percent). The patients spent an average of 9.3 (8.1) days in the hospital in the robotic group and 9.2 (6.8) days in the laparoscopic group (P=.79). The distal resection margin (P =.003), as well as the number of lymph nodes collected in the specimen (P =.23), were both higher in the robotic surgery specimen.  Conclusion: Robotic colorectal surgery was conducted safely and successfully, and the clinical outcomes were comparable. |
| [42] | Jayne et al; 2017; multicentric; completed | Effect of robotic assisted vs conventional laparoscopic surgery on risk of conversion to open laparotomy among patients undergoing resection for rectal cancer the rolarr randomized clinical trial | 471 patients with rectal adenocarcinoma involved in a randomized clinical trial | Patients were randomly assigned to robotic assisted (n = 237) or traditional (n = 234) laparoscopic rectal cancer resection, with either high (upper rectum) or low (whole rectum) anterior resection or abdominoperineal resection as the procedure of choice (rectum and perineum).  Conversion to open laparotomy was the major outcome. Intraoperative and postoperative complications, circumferential resection margin positivity (CRM+) and other pathological outcomes, quality of life (36-Item Short Form Survey and 20-item Multidimensional Fatigue Inventory), bladder and sexual dysfunction (International Prostate Symptom Score, International Index of Erectile Function, and Female Sexual Function Index), and oncological outcomes were among the secondary end points. | | The overall rate of open laparotomy conversion was 10.1 percent, with 19 of 236 patients (8.1 percent) in the robotic-assisted laparoscopic group and 28 of 230 patients (12.2%) in the conventional laparoscopic group (unadjusted risk difference = 4.1 percent [95 percent CI, -1.4 percent to 9.6 percent]; adjusted odds ratio = 0.61 [95 percent CI, 0.31 to 1.21]; P =.16). CRM+ was found in 14 (6.3%) of 224 patients in the conventional laparoscopic group and 12 (5.1%) of 235 patients in the robotic-assisted laparoscopic group (unadjusted risk difference = 1.1 percent [95 percent CI, -3.1 percent to 5.4 percent]; adjusted odds ratio = 0.78 [95 percent CI, 0.35 to 1.76]; P =.56). None of the other eight predetermined secondary end objectives, such as intraoperative problems, postoperative complications, plane of surgery, 30-day mortality, bladder dysfunction, and sexual dysfunction, revealed a statistically significant difference between groups.  Conclusion: When performed by surgeons with varied levels of robotic surgery experience, robotic-assisted laparoscopic surgery does not provide an advantage in rectal cancer resection. |
| [43] | Park et al; 2012; USA; completed | Reverse-hybrid robotic mesorectal excision for rectal cancer | 30 rectal cancer patients involved | From January 2009 to March 2011, patients who underwent robotic rectal cancer resection were enrolled in this prospective cohort observational study. A technique for reverse-hybrid robotic-laparoscopic rectal resection with radical lymphadenectomy was developed throughout the study period. This technique involves reversal of the operative sequence with lymph vascular and rectal dissection to precede proximal colonic mobilization. This technique evolved from a conventional-hybrid resection with laparoscopic vascular control, colonic mobilization, and robotic pelvic dissection. Perioperative and short-term oncologic outcomes were analysed. | | Reverse-hybrid resection was performed on thirty patients. The median tumour was 5 cm from the anal edge (interquartile range 3-9). The median BMI was 27.6 kilograms per meter squared (interquartile range: 25.0-32.1 kilograms per meter squared). Neoadjuvant chemoradiation was given to 20 (66.7%) of the patients. No conversions were made. The average amount of blood lost was 100 mL. (Interquartile range 75-200). The median surgery time was 369 minutes (interquartile range 306-410). The median docking time was 6 minutes (interquartile range: 5-8), while the median console time was 98 minutes (interquartile range: 88-140). All patients had R0 resections; no one had an incomplete mesorectal resection. Six patients (20%) had en bloc resection or extensive lymph node dissection.  Conclusions: For optimal application in minimally invasive rectal surgery, reverse-hybrid robotic surgery for rectal cancer maximizes the therapeutic applicability of robotic and conventional laparoscopic procedures. |
| [44] | Colombo et al; 2015; France; completed | Robotic Versus Laparoscopic Total Mesorectal Excision (TME) for SphincterSaving Surgery: Is There Any Difference in the Transanal TME Rectal Approach? : A Single-Centre Series of 120 Consecutive Patients | 120 rectal cancer patients involved | A total of 120 rectal carcinomas were enrolled in the study, all of which were operated on for a sphincter-saving operation. The study covered the first 60 robotic surgery and the last 60 laparoscopic surgeries (six hybrid approaches, then 54 full robotic surgeries). | | Patients in both the R-TME and L-TME groups had similar baseline characteristics. Blood loss (200 vs. 100 mL), postoperative hospital stays (12 vs. 11 days), conversion rate (3.2 vs. 4.8%), lymph nodes yield (15 vs. 19), no positive distal margin (0%), positive radial margin (6.4 vs. 9.3%), diverting ileostomy (73 vs. 58%), and severe morbidity (73 vs. 58%) were all comparable (28 vs. 20 percent). Proctectomy performed through transanal route (1.7 vs. 16.7%; p = 0.004) had significant variations in median operative time (274 vs. 228 min; p = 0.003) and median operative time (274 vs. 228 min; p = 0.003). After the first 25 procedures, the R-TME operative time curve stabilized at 245 minutes.  Conclusion: In terms of methodology, R-TME may be as practical and safe as L-TME for rectal cancer. R-TME provides complete rectal dissection with an abdominal approach in our practice and for tough cases, whereas L-TME necessitates a transanal approach. |
| [45] | Van Loon et al; 2014; The Netherlands; completed | Outcome of transoral robotic surgery for stage I–II oropharyngeal cancer. Eur Arch Oto-Rhino- Laryngology | 18 oropharyngeal carcinoma patients were included | The Netherlands Cancer Institute used the da Vinci robot system to perform transoral resection on 8 patients with early-stage oropharyngeal tumours. The self-report ratings of quality-of-life questionnaires were completed by all surviving patients. The median time for a robot-assisted operation was 115 minutes (range 43-186 minutes), with a 5 ml median estimated blood loss (range 0-125 ml). | | Because to tumour extension and local anatomy in three cases, the exposure was insufficient to acquire clean tumour margins. Fourteen of the patients had surgical margins that were completely clean. Adjuvant radiation was given to four patients. An elective unilateral neck dissection was performed on nine patients. In two cases, oropharyngeal cancer recurred. Patients who required postoperative radiation fared worse in terms of quality of life, while those who underwent transoral resection fared well. Based on this small sample of patients, TORS appears to be an oncologically safe surgical treatment for early stage T1-2N0 oropharyngeal cancer. |
| [46] | Gupta et al; 2010; India; completed | Robot-assisted Adrenal-Sparing Surgery for Pheochromocytoma: Initial Experience | 4 adrenal cancer patients involved | Standard preoperative preparation was performed on four patients with metabolically active adrenal pheochromocytoma (two right, two left). All of the cases were treated with a robotic transperitoneal technique. In every case, the surrounding normal parenchyma was spared. All pertinent perioperative data was gathered and examined. | | The average operational time was 77.5 minutes (range 40-140 minutes), with a 97.5 mL blood loss (range 50-160 mL). There were no perioperative problems or conversions. The average tumour size was 4.7 cm in diameter. There were three instances of intraoperative hypertension that required treatment. The average dose of diclofenac was 150 mg, and patients were allowed to consume it orally after 6 hours. The drain was removed within 24 hours, and the patient spent an average of four days in the hospital. The histopathology report confirmed pheochromocytoma in all patients with free surgical resection margins. There was no sign of recurrence or extra-adrenal tumour after an average follow-up of 9 months (range 4-14 months).  Conclusion: In our preliminary experience, robot-assisted pheochromocytoma excision is feasible, safe, and effective. |
| [47] | Phee et al; 2012; multicentric; completed | Robot-Assisted Endoscopic Submucosal Dissection Is Effective in Treating Patients With Early- Stage Gastric Neoplasia | 5 gastrointestinal cancer patients involved | Five individuals with early-stage gastric neoplasia restricted to the mucosa were studied in a multicentre prospective investigation. All submucosal dissections were done with the MASTER system after marks and circumferential mucosal incision. We looked at demographics at the start, tumour features, and perioperative and clinical outcomes. | | The average time it took to dissect the submucosa was 18.6 minutes (median 16 minutes; range 3–50 minutes). There were no perioperative problems. After the surgeries, three patients were discharged from the hospital within 12 hours and two on the third day. Two individuals had intramucosal adenocarcinoma, one had high-grade dysplasia, one had low-grade dysplasia, and one had a hyperplastic polyp, according to the findings. In all five cases, the resection margins were free of tumours. The 30-day follow-up check-up revealed no problems. Endoscopic investigations found no remaining or recurring malignancies in any of the individuals.  Conclusion: ESD may be performed with a flexible endoscopic robotic system, and patients with early gastric neoplasia can be efficiently treated. |
| [48] | Weizer et al; 2011; USA; completed | Robot-assisted retroperitoneal partial nephrectomy: Technique and perioperative results | A single case renal cancer assessed | The robot-assisted retroperitoneal partial nephrectomy procedure was successfully completed. | | The procedure was safe and effective and overall outcomes were excellent |
| [49] | Van der Sluis; 2019; The Netherlands; completed | Robot-assisted minimally invasive thoraco-laparoscopic esophagectomy versus open transthoracic esophagectomy for resectable esophageal cancer, a randomized controlled trial (ROBOT trial) | 112 patients with resectable intrathoracic oesophageal cancer | In a single centre randomized controlled trial, 112 patients with resectable intrathoracic oesophageal cancer were randomly assigned to either RAMIE or OTE. The primary outcome was the occurrence of postoperative surgery-related complications in general (modified Clavien-Dindo classification grade 2-5). | | Overall, RAMIE (59%) was associated with fewer surgery-related postoperative problems than OTE (80%) [risk ratio with RAMIE (RR) 0.74; 95 percent confidence interval (CI), 0.57-0.96; P = 0.02]. When compared to OTE, RAMIE resulted in less median blood loss (400 vs 568 mL, P 0.001), a lower percentage of pulmonary (RR 0.54; 95 percent CI, 0.34-0.85; P = 0.005) and cardiac (RR 0.47; 95 percent CI, 0.27-0.83; P = 0.006) complications, and lower mean postoperative pain (visual analog scale, 1.86 vs 2.62; P 0.001). The RAMIE group had a greater functional recovery at postoperative day 14 [RR 1.48 (95 percent CI, 1.03-2.13; P = 0.038)]. with a higher discharge quality of life score [mean difference quality of life score 13.4 (2.0-24.7, p = 0.02)] and 6 weeks after discharge [mean difference 11.1 (1.0-21.1; P = 0.03) quality of life score]. At a mean follow-up of 40 months, short- and long-term oncological results were equivalent.  Conclusion: When compared to OTE, RAMIE resulted in a lower percentage of total surgery-related and cardiopulmonary problems, as well as lower postoperative pain, a higher short-term quality of life, and a better short-term functional recovery. Oncological results were comparable and in line with today's top standards. |

| **Articles on AI applicability in the treatment of cancer focusing on clinical decision making** | | | | | |
| --- | --- | --- | --- | --- | --- |
| **Ref** | **Author(s), year, location, status** | **Title** | **Population** | **Intervention** | **Outcomes** |
| [50] | Ko et al; 2018; Taiwan; completed | Clinically validated machine  learning algorithm for detecting residual diseases with multicolor flow cytometry  analysis in acute myeloid leukemia and myelodysplastic syndrome | 1742 patients suffering from myeloid leukaemia and myelodysplastic syndrome | The researchers gathered 5333 MFC data from 1742 AML or MDS patients. For clinical outcome validation, the 287 MFC data at post-induction were chosen as the outcome set. The rest were divided into two groups: training (n = 4039) and validation (n = 1007). The performance of the AI algorithm was assessed in the validation set after it learned a multi-dimensional MFC phenotype from the training set and input it to a support vector machine (SVM) classifier after Gaussian mixture model (GMM) modelling. | The developed algorithms produced promising accuracies (84.6% to 92.4%) and AUCs (0.921-0.950). Surprisingly, the algorithm from just one testing tube performed similarly. The clinical significance was confirmed in the outcome set, with normal MFC interpreted by AI predicting higher progression-free survival (10.9 vs 4.9%, p 00001) and overall survival (13.6 vs 6.5%, p 00001) for AML patients. |
| [51] | Skerede et al; 2020; multicentric; completed | Deep learning for prediction of colorectal cancer outcome: a discovery and validation study | 920 colorectal cancer patients involved | A total of ten convolutional neural networks, purpose-built for categorizing supersized heterogeneous images, were trained using more than 12 000 000 image tiles from patients with a distinguishing favourable or poor illness outcome from four cohorts. Patients with a non-distinct outcome were used to develop a prognostic biomarker that integrated the ten networks. The marker was tested on 920 individuals with slides prepared in the United Kingdom, and then independently validated in 1122 patients treated with single agent capecitabine using slides prepared in Norway, according to a predetermined procedure. Only patients with resectable tumours were included in each cohort, as well as a formalin-fixed, paraffin-embedded tumour tissue block for examination. Cancer-specific survival was the primary endpoint. | 828 patients from four cohorts were employed as a training cohort to acquire clear ground truth. Tuning was done on 1645 patients who had a non-distinct outcome. In the primary analysis of the validation cohort, the biomarker had a hazard ratio of 384 (95 percent CI 272–543; p00001) for poor versus good prognosis, and a hazard ratio of 304 (207–447; p00001) after adjusting for established prognostic markers significant in univariable analyses of the same cohort, which were pN stage, pT stage, lymphatic invasion, and venous |
| [52] | Wagner at al; 2019; The Netherlands; completed | .A parsimonious 3-gene signature predicts clinical outcomes in an acute myeloid leukemia multicohort study | 593 adults with no promyelocytic acute myeloid leukaemia | On a publicly available data set for a discovery cohort of 593 people with no promyelocytic AML, we used an artificial neural network (ANN)-based machine learning approach. CALCRL, CD109, and LSP1 were discovered as a parsimonious 3-gene expression signature that was predictive of event-free survival (EFS) and overall survival (OS) using ANN analysis (OS). We constructed a prognostic index (PI) by combining clinically validated prognosticators with normalized gene-expression levels and -values from Cox proportional hazards models. Patients with very high-risk features, such as a high PI and either FLT3 internal tandem duplication or nonmutated nucleophosmin 1, were identified using our 3-gene PI, which divided adult patients in each European LeukemiaNet cytogenetic risk category into subgroups with different survival probabilities. | After controlling for recognized prognosticators, the PI remained significantly linked with poor EFS and OS, and its capacity to stratify survival was validated in three independent adult cohorts (n = 905 participants) and one juvenile AML cohort (n = 145 subjects). In addition, in silico investigations revealed that AML was the only tumour type out of 39 that had contemporaneous overexpression of CALCRL, CD109, and LSP1 that predicted survival. As a result, our ANN-derived 3-gene signature improves patient stratification accuracy and has the potential to improve outcome prediction dramatically. |
| [53] | Dai et al; 2018; USA; completed | Case-only Methods Identified Genetic Loci Predicting a Subgroup of Men With Reduced Risk of High-grade Prostate Cancer by Finasteride | 1157 prostate cancer patients involved | Case-only approaches were used to evaluate germline genomic data from 1,157 prostate cancer cases in PCPT. 357 SNPs from 83 potential genes in androgen metabolism, inflammation, circadian rhythm, and other pathways were included in the genotypes. Individual SNPs were tested in a univariate case-only analysis to see if they changed the effect of finasteride on the incidence of high-grade and low-grade prostate cancer. To find a predictive signature for genotype-specific treatment effects, researchers used case-only classification trees and random forests, which are sophisticated machine learning approaches with resampling-based controls for model complexity. A single SNP in the SRD5A1 gene (rs472402) significantly affected the finasteride effect on high-grade prostate cancer (Gleason score > 6) in PCPT (family-wise error rate 0.05) after accounting for repeated testing. | When men with the GG genotype at this locus were given finasteride, their chance of developing high-grade cancer fell by 55 percent (RR = 0.45; 95 percent confidence interval, 0.27-0.75). Case-only trees and random forests were used to find additional effect-modifying SNPs with moderate statistical significance. When using the case-only random forest method with 28 chosen SNPs, a prediction model found that 37% of PCPT males have a lower chance of high-grade prostate cancer when using finasteride, whereas the remainder have a higher risk. |
| [54] | Zhong et al; 2018; China; completed | A response prediction model for taxane, cisplatin, and 5-fluorouracil chemotherapy in hypopharyngeal carcinoma | 29 patients suffering from head and squamous cell carcinoma were involved | TPF-sensitive and non-sensitive patient samples have their gene expression profiles examined. Between these two groups, we discovered a gene expression signature. We then selected 10 genes and used a support vector machine (SVM) model to train it. | To predict the response to TPF treatment in our patients, this model has an 88.3 percent sensitivity and an 88.9 percent specificity. For additional validation, four more TPF responsive and four more TPF non-sensitive patient samples were employed. This SVM model has been shown to have a sensitivity of 75.0 percent and a specificity of 100 percent in predicting TPF response in new patients. This shows that our 10-gene SVM prediction model could help doctors personalize HNSCC treatment for patients. |
| [55] | Wan et al; 2012; China; completed | Molecular prognostic prediction for locally advanced nasopharyngeal carcinoma by support vector machine integrated approach | 97 patients suffering from nasopharyngeal carcinoma were involved | A total of 97 patients with locally progressed NPC were evaluated in a randomized controlled trial (RCT) with a 5-year follow-up, with 48 cases serving as the training set and 49 cases acting as the testing set of SVM models. SVM models were created by selecting variables from 38 tissue molecular biomarkers representing six cancer signalling pathways and three EBV-related serological biomarkers. With a 5-year follow-up, we developed three SVM models to refine NPC prognosis. | By combining the expression of seven molecular biomarkers, the SVM1 demonstrated strong prediction sensitivity (sensitivity and specificity were 88.0 percent and 81.9 percent, respectively). By aggregating the expression levels of 12 molecular biomarkers and three EBV-related serological biomarkers, the SVM2 model demonstrated strong predictive specificity (sensitivity and specificity were 84.0 percent and 94.5 percent, respectively). The SVM3 model, which was built by combining SVM1 and SVM2, had a strong predictive capacity (sensitivity, specificity were 88.0 percent and 90.3 percent, respectively). Three SVM models were shown to have high power in prognostic categorization. Furthermore, Cox multivariate regression analysis revealed that these three SVM models were all meaningful independent prognostic models for overall survival in both the testing set and the overall patients.  Conclusion: In the RCT, our SVM prognostic models performed well in refining patient prediction for locally progressed NPC, potentially guiding future target therapy against associated signalling pathways. |
| [56] | Niikura et al; 2020; Japan; completed | Artificial Intelligence Versus Expert Endoscopists for Diagnosis of Gastric Cancer | 500 gastric cancer patients involved | We used data from 500 patients, including 100 with stomach cancer, who were matched 1:1 to AI or professional endoscopists' diagnoses. We compared the per-image rate of gastric cancer diagnosis with the non-inferiority (prespecified margin of 5%) of the per-patient rate of gastric cancer diagnosis by AI. | Gastric cancer was identified in 49 of 49 AI patients (100%) and 48 of 51 expert endoscopist patients (94.12%). (Difference 5.88, 95 percent confidence interval: -0.58 to 12.3). The AI group had a higher per-image rate of stomach cancer diagnosis (99.87 percent, 747/748 images) than the expert endoscopist group (88.17 percent, 693/786 images) (difference 11.7 percent)  Conclusion: There was no evidence of inferiority in the rate of stomach cancer diagnosis by AI, but there was no evidence of superiority. |
| *  [57] | Zui et al; China; not yet completed | Development and Validation of a Deep Learning Algorithm for Bowel Preparation Quality Scoring | 100 colorectal cancer patients | The goal of this study is to develop an algorithm to assess bowel preparation quality using the BBPS and see if AI may help enhance colonoscopy quality control parameters. |  |
| *  [58] | Kurnaz S, Johnston K; ESTIMATED 2022; USA; not yet completed | SYNERGY-AI: Artificial Intelligence Based Precision Oncology Clinical Trial Matching and Registry | 5000 participants | Patients freely join the registry, which is non-interventional and does not need any protocol-mandated tests or procedures. All treatment decisions are done at the discretion of the PP in collaboration with their patients, using the AI CT matching report and VTB support. CTE will be evaluated based on biomarkers and enrolment hurdles, among other things. The study will last 36 months (24 months of enrolment followed by 12 months of data collecting, which will happen every three months). The primary analysis will take place 12 months after the final patient has been enrolled. |  |
| *  [59] | Honggang Yet al; estimated 2021; China | A Single Center Study on the Effectiveness and Safety of Polyp Classification With Artificial Intelligence | Estimated 70 patients with presence of malignant polyp | The goal of the study is to use AI to help clinicians diagnose polyps and improve the quality of training. |  |
| *  [60] | Chen et al; estimated 2021; Taiwan; not yet completed | Computer-aided Detection for Colonoscopy | Estimated 1000 colorectal patients | The goal of this study is to compare computer-assisted colonoscopy versus traditional colonoscopy in a randomized clinical trial. |  |

**Studies not yet completed or forthcoming*

|  |  | | **Table C1** Articles on AI applicability in the treatment of cancer focusing on clinical imaging techniques | | | | | | | | |
| --- | --- | --- | --- | --- | --- | --- | --- | --- | --- | --- | --- |
| Reference | | Year | | Country | Type of cancer | Number of patients | Type of study | Control group | Experimental group | 95% CI reported | Average follow-up period/follow-up checkpoints (months/years) |
| Sayesteh et al.  [17] | | 2019 | | Iran | Rectal cancer | 98 | - | - | - | No | - |
| De Jong et al.  [18] | | 2018 | | Multicentric | Non-squamous cell carcinoma | 1^st^ cohort: 285  2^nd^ cohort: 223 | 1^st^ cohort: interventional  2^nd^ cohort: interventional, randomized allocation | 112 | 111 | Yes | 1^st^ cohort: 52 months  2^nd^ cohort: 28 months |
| Xiong et al.  [19] | | 2018 | | China | Oesophageal squamous cell carcinoma | 30 | Interventional | - | - | Yes | 1 month after completion; 3 months every 3 months and every 6 months thereafter |
| Zhao et al.  [20] | | 2010 | | USA | Breast cancer | 400 | - | - | - | No | - |
| Chen et al.  [21] | | 2013 | | USA | Lung cancer | 11 | - | - | - | No | - |
| * Zhao et al.  [22] | | Estimated 2024 | | China | Hepatocellular carcinoma | Estimated 1200 | Observational | - | - | - | - |
| *Whan et al.  [23] | | Estimated 2021 | | China | Rectal cancer | Estimated 205 | Observational | - | - | - | - |
| *Juneau et al.  [24] | | Estimated 2024 | | Canada | Prostate cancer | Estimated 1000 | Interventional | - | - | - | - |
| *Zhang et al.  [25] | | Estimated 2025 | | China | Recurrent gliomas | Estimated 600 | Observational | - | - | - | Target: 36 months |
| *Xin et al.  [26] | | Estimated 2022 | | China | Gliomas | Estimated 350 | Observational | - | - | - | - |
| *Xu et al.  [27] | | Estimated 2022 | | China | Cervical cancer | Estimated 122 | Interventional, randomized allocation | - | - | - | - |
| *Wing et al.  [28] | | Estimated 2024 | | China | Breast cancer | Estimated 1000 | Observational | - | - | - | - |
| *Mutter et al.  [29] | | Estimated 2021 | | France | Colorectal cancer | Estimated 34 | Interventional |  |  |  |  |

**Studies not yet completed or forthcoming*

|  |  | | **Table C2** Articles on AI applicability in the treatment of cancer focusing on robotic surgery | | | | | | | | |
| --- | --- | --- | --- | --- | --- | --- | --- | --- | --- | --- | --- |
| Reference | | Year | | Country | Type of cancer | Number of patients | Type of study | Control group | Experimental group | 95% CI reported | Average follow-up period/follow-up checkpoints (months/years) |
| Asimakapolous et al.  [30] | | 2011 | | USA | Prostate cancer | 128 | Interventional, randomized allocation | 64 | 64 | No | 12 months |
| Engel et al.  [31] | | 2011 | | Multicentric | Prostate cancer | 127 | Interventional, randomized allocation | - | - | No | 11 months |
| Pierro et al.  [32] | | 2011 | | Switzerland | Prostate cancer | 150 | - | - | - | No | 3 & 12 months |
| Porpiglia et al.  [33] | | 2013 | | Italy | Prostate cancer | 120 | Interventional, randomized allocation | 60 | 60 | No | 3 & 12 months |
| Tan et al.  [34] | | 2016 | | USA | Prostate cancer | 48 | - | - | - | No | 2 days, 2 weeks, 2 months |
| Chang et al.  [35] | | 2018 | | Korea | Prostate cancer | 17 | - | - | - | No | 3 months |
| Shah & Abazza.  [36] | | 2011 | | USA | Prostate cancer | 100 | - | - | - | No |  |
| Chung et al.  [37] | | 2011 | | Korea | Prostate cancer | 265 | Interventional, randomized allocation | 105 | 155 | No | 12 months |
| Nix et al.  [38] | | 2010 | | USA | Bladder cancer | 41 | Interventional, randomized allocation | 21 | 20 | Yes | - |
| Richards et al.  [39] | | 2011 | | USA | Bladder cancer | 60 |  |  |  | No | Every 3 months for one year & every 6 months for year two and three |
| Parekh et al.  [40] | | 2013 | | USA | Bladder cancer | 47 | Interventional, randomized allocation |  |  | No |  |
| Jimenez et al.  [41] | | 2011 | | Spain | Colorectal cancer | 56 | Interventional, randomized allocation |  |  | No |  |
| Jayne et al.  [42] | | 2017 | | Multicentric | Rectal adenocarcinoma | 471 | Interventional, randomized allocation | 237 | 234 | Yes | 1 month, 6 months, 12 months |
| Park et al.  [43] | | 2012 | | USA | Rectal cancer | 30 | Observational | - | - | No | - |
| Colombo et al.  [44] | | 2015 | | France | Rectal cancer | 120 | - | - | - | No | Every 3 months for 3 years, then every 6 months until 5^th^ year |
| Van Loon et al.  [45] | | 2014 | | The Netherlands | Oropharyngeal carcinoma | 18 | - | - | - | No | 6-68 months |
| Gupta et al.  [46] | | 2010 | | India | Adrenal cancer | 4 | - | - | - | No | - |
| Phee et al.  [47] | | 2012 | | multicentric | Gastrointestinal cancer | 5 | Interventional | - | - | No | 1mo |
| Weizer et al.  [48] | | 2011 | | USA | Renal cancer | 1 | - | - | - | No | - |
| Van der Sluis et al.  [49] | | 2019 | | The Netherlands | Intrathoracic oesophageal cancer | 112 | Interventional, randomized allocation | 56 | 56 | Yes | Medium 40 months |

**Studies not yet completed or forthcoming*

|  |  | | **Table C3** Articles on AI applicability in the treatment of cancer focusing on clinical decision making | | | | | | | | |
| --- | --- | --- | --- | --- | --- | --- | --- | --- | --- | --- | --- |
| Reference | | Year | | Country | Type of cancer | Number of patients | Type of study | Control group | Experimental group | 95% CI reported | Average follow-up period/follow-up checkpoints (months/years) |
| Ko et al.  [50] | | 2018 | | Taiwan | Myeloid leukaemia & myelodysplastic syndrome | 1742 | Interventional, randomized allocation | 4039 | 1007 | Yes | 21.3 months average (1-96.1) |
| Skerede et al.  [51] | | 2020 | | Multicentric | Colorectal cancer | 920 | Interventional | - | - | Yes | - |
| Wagner et al.  [52] | | 2019 | | The Netherlands | No promyelocytic acute myeloid leukaemia | 593 | - | - | - | Yes | Average between 5 groups: 5,65 years |
| Dai et al.  [53] | | 2018 | | USA | Prostate cancer | 1157 | Observational | - | - | Yes | 7 years |
| Zhong et al.  [54] | | 2018 | | China | Head and squamous cell carcinoma | 29 |  |  |  | No |  |
| Wan et al.  [55] | | 2012 | | China | Nasopharyngeal carcinoma | 97 | Interventional, randomized allocation | 48 | 49 | Yes | 5 years |
| Niikura et al.  [56] | | 2020 | | Japan | Gastric cancer | 500 retrospective data and 100 gastric patients | 100 patients: interventional | - | - | Yes | - |
| *Zui et al.  [57] | | - | | China | Colorectal cancer | Estimated 100 | Interventional, randomized allocation | - | - | - | - |
| *Kurnaz et al.  [58] | | Estimated 2022 | | USA | Advanced solid and haematological malignancies | Estimated 5000 | Observational |  |  |  | Target 36 months |
| *Honggang et al.  [59] | | Estimated 2021 | | China | Malignant polyps | Estimated 70 | Observational | - | - | - | - |
| *Chen et al.  [60] | | Estimated 2021 | | Taiwan | Colorectal patients | Estimated 1000 | Interventional, randomized allocation | - | - | - | - |

**Studies not yet completed or forthcoming*
